# Determinants of Infant and Young Child Feeding Practices of Children With Severe Acute Malnutrition in Agrarian and Pastoralist Settings of Ethiopia

**DOI:** 10.1111/mcn.70217

**Published:** 2026-07-05

**Authors:** Mohammed Areb, Lieven Huybregts, Dessalegn Tamiru, Mariama Touré, Bayise Biru, Talla Fall, Alemayehu Haddis, Tefera Belachew

**Affiliations:** ^1^ Department of Nutrition and Dietetics, Institute of Health Sciences Faculty of Public Health Jimma University Jimma Oromia Ethiopia; ^2^ Haramaya University Maya Oromia Ethiopia; ^3^ Nutrition, Diets, and Health Unit International Food Policy Research Institute (IFPRI) Washington District of Columbia USA; ^4^ International Food Policy Research Institute Nutrition, Diets, and Health Unit Dakar Senegal; ^5^ Institute of Health Sciences, School of Public Health, Nekemte Wallaga University Ethiopia; ^6^ Department of Environmental Science and Technology Jimma University Jimma Ethiopia; ^7^ Ethiopian Public Health Association Addis Ababa Ethiopia; ^8^ Ethiopian Midwives Association Addis Ababa Ethiopia

**Keywords:** agrarian and pastoralist settings, egg and flesh food consumption, Ethiopia, infant and young child feeding, minimum dietary diversity, minimum meal frequency, severe acute malnutrition

## Abstract

Previous research has established determinants of young child feeding (IYCF) practices in the general child population, but determinants of these practices in children with severe acute malnutrition (SAM) are unknown. This study assessed the determinants of IYCF practices among children aged 6–23 months with SAM and under SAM treatment in a pastoralist and a predominantly agrarian setting in Ethiopia. As part of the baseline survey for the R‐SWITCH cluster randomized control trial, we screened ~28,000 under‐fives and included 486 children aged 6–23 months with SAM and under SAM treatment. Multivariable regression analysis was used to identify determinants on household, caregiver and child levels. Minimum meal frequency (MMF) was similar between children from the agrarian (53.9%) and pastoralist (51.9%) setting. The prevalence of children with minimum dietary diversity (MDD) was low overall but higher among pastoralist children (31.5%) than children from the agrarian setting (19.0%; *p* = 0.012). The consumption of egg/flesh foods was lower among pastoralist children (5.6%) than agrarian children (16.9%, *p* = 0.085). Caregiver literacy was positively associated with a higher likelihood of MMF (IRR = 1.21; 95% CI:1.02–1.43; *p* = 0.030), while caregiver depression was associated with a lower likelihood of MMF (RR = 0.97; 95% CI:0.95–0.99; *p* = 0.003). Improved water source (RR = 1.59; 95% CI:1.06–2.38; *p* = 0.037), caregiver literacy (RR = 2.04; 95% CI:1.25–3.34; *p* = 0.002), caregiver MDD (RR = 2.21; 95% CI:1.60–3.05; *p* ≤ 0.001), older children(RR = 1.05; 95% CI: 1.02–1.08;*p* ≤ 0.001), and pastoralist setting (RR = 1.62; 95% CI:1.07–2.44; *p* = 0.014) were associated with a significantly higher likelihood of meeting MDD. Conversely, caregiver depression (RR = 0.94; 95% CI:0.90–0.97; *p* < 0.001), caregiver mobility restriction (IRR = 0.94; 95% CI:0.89–1.00; *p* = 0.025), and food insecurity (RR = 0.64; 95% CI:0.41–1.01; *p* = 0.05) were associated with reduced MDD likelihood. Caregiver community involvement (IRR = 1.12; 95% CI:1.00–1.26; *p* = 0.038), caregiver MDD (RR = 1.67; 95% CI:1.10–2.53; *p* = 0.043), and older children (RR = 1.08; 95% CI:1.05–1.12; *p* < 0.001) were associated with increased egg/flesh‐food consumption among children, while living in a pastoralist setting was associated with reduced it (RR = 0.36; 95% CI:0.13–0.97; *p* = 0.038). Integrated and Targeted interventions recommended: caregiver literacy/depression support to improve IYCF; livelihood‐specific foods (eggs/flesh, fruits/vegetables); WaSH enhancements; community engagement; and IYCF counseling within CMAM programs.

## Summary

- This study examined determinants of infant and young child feeding practices among children 6–23 months old with severe acute malnutrition in Ethiopia.
- Minimum meal frequency was similar in agrarian and pastoralist settings, but minimum dietary diversity was low overall.
- Caregiver literacy, water access, caregiver dietary diversity, and older child age were associated with better feeding practices.
- Caregiver depression, mobility restriction, and food insecurity were associated with poorer feeding practices.
- The findings support integrated interventions, including caregiver support, improved food access, WaSH improvements, community engagement, and IYCF counseling in CMAM programs.

## 1 Introduction

Children from 6 months require complementary foods in addition to breastfeeding, which should continue up to 23 months or beyond (Lutter et al. 2021). Studies show improved dietary diversity, higher meal frequency, and consumption of animal source foods (ASF) are linked to better child nutrition and growth (Arimond and Ruel 2004; Bonis‐Profumo et al. 2021; Hanley‐Cook et al. 2022). In 2008, UNICEF and the World Health Organization (WHO) developed infant and young child feeding (IYCF) indicators to improve the assessment of complementary feeding practices (WHO 2010). Three core indicators of minimum dietary diversity (MDD), minimum meal frequency (MMF), and minimum acceptable diet (MAD) evaluate complementary feeding in children aged 6–24 months. These indicators correlate with better energy and micronutrient intake and improved growth (WHO/UNICEF 2021).

Although these indicators are routinely collected in the general child population by many national surveys such as the Demographic Health Surveys and the Multiple Indicator Cluster Surveys, less is known about the variation and factors associated with IYCF practices within children with SAM and under SAM treatment.

This community‐based study evaluated multiple World Health Organization IYCF indicators among children aged 6–23 months with severe acute malnutrition (SAM), including those undergoing treatment and those in recovery. The study was conducted across pastoralist and agrarian communities in Ethiopia and was designed to ensure a population‐representative sample that reflects the diverse characteristics of children, caregivers, and households accessing care (Shafiq et al. 2013). Existing research either relies on SAM cases enrolled from treatment sites which are a sample unlikely to be representative for the population (Lenters et al. 2013), or fails to account for nutritional status or lacks disaggregation by treatment context an important gap (Golden and Grellety 2007; World Health Organization 2007), given that this approach addresses a critical gap by linking IYCF indicators to both the onset and recovery from SAM (Black et al. 2013; Long et al. 2024; WHO 2010). In this study we first assessed the prevalence of IYCF indicators among children aged 6–23 months with SAM and under SAM treatment or recovering from SAM in agrarian and pastoralist settings in Ethiopia. We then identified factors associated with IYCF indicators at the household, caregiver, and child levels.

## 2 Methods and Materials

### 2.1 Study Design & Setting

The data for this study were obtained from the baseline survey of R‐SWITCH Ethiopia that was conducted as part of a cluster randomized controlled trial (cRCT) evaluating the impact of intervention. The R‐SWITCH intervention is an integrated package designed to enhance SAM detection and treatment coverage across the full continuum of care prevention, screening, referral, treatment, and relapse prevention at household, community, and facility levels in two Ethiopian woredas. It features monthly group behavior change communication by Alliance for Development (AFD) community groups on IYCF, health, and Water, Sanitation and Hygiene (WaSH) with food‐based recipe demonstrations; weekly family‐led MUAC screening by caregivers plus active/passive screening by AFD and health services; expanded admission criteria (mid‐upper arm circumference (MUAC) < 115 mm, edema, or Weight‐for‐age Z‐score (WAZ) < −3). The intervention was delivered via health posts, AFD groups, and community leaders as part of a cRCT design. The survey included 486 children aged 6–23 months who were either suffering from SAM or receiving treatment for it. Data were collected from 40 health post catchment areas across two contrasting livelihood zones: (i) agrarian Kersa in Jimma Zone, Oromia Region, and (ii) pastoralist settings of Jeldesa in Dire Dawa city administration. According to Ethiopian Standardized Monitoring and Assessment of Relief and Transitions (SMART) data 2019–2022, SAM prevalence among under‐fives in Oromia was 3.9%, slightly higher than the 3.5% seen in other Ethiopian regions. Each region's woredas were chosen based on security, livelihood diversity, accessibility, and SAM caseload. Kersa (~357 km Southwest of Addis) is a rural, coffee‐growing woreda with 40,567 under‐fives, while Jeldesa (3,956 under‐fives) typifies pastoralist and agrarian systems. Jeldesa woreda is significantly smaller than Keresa woreda, explaining the smaller pastoralist sample size from study outset. Of a total of 44 health posts/kebeles, 40 eligibles (with ≥ 2 Health Extension workers (HEWs)) across both woredas participated in the survey. Woredas are key administrative districts, while kebeles are the smallest, community‐level units responsible for basic services, security, and development.

Enumerator teams conducted door‐to‐door visits to perform anthropometric measurements on all children aged 6–59 months across both woredas. Children identified SAM, defined as a MUAC < 115 mm, a weight‐for‐length Z‐score (WLZ) < –3 based on the 2006 WHO growth standards, bilateral pitting edema, or those enrolled in the existing SAM Outpatient Therapeutic Program (OTP) were considered eligible for the study. Child enrolled in SAM OTP was based on three criteria: (i) the caregiver reporting that the child was enrolled in SAM OTP at the time of the survey, (ii) the caregiver reporting that the child was given RUTF in the last 3 days, and (iii) the caregiver able to show at least in full sachet of RUTF or two or more empty RUTF sachets. Children with congenital conditions that precluded accurate anthropometric measurement, as well as those whose caregivers did not provide consent, were excluded.

### 2.2 Sample Size

The sample size was determined as part of the R‐SWITCH Ethiopia baseline survey for a cRCT, using estimates of SAM OTP treatment coverage among children aged 6–59 month. A conservative treatment coverage estimate of 50% was used due to limited recent data. To detect a 15% increase in treatment coverage (from 50% to 65%) with 90% power, 5% significance, a design effect of 2, and a cluster size of 30 (accounting for 10% non‐response), 40 clusters (health posts) were required. After adjusting for 10% non‐response, the sample included 684 children with SAM and under SAM treatment or receiving OTP. As part of the R‐SWITCH Ethiopia cRCT baseline survey, all eligible children aged 6–23 months with SAM and under SAM treatment or enrolled in SAM OTP (*n* = 486) of the two study woredas were included in this study from May to September 2024. Using Survey Solutions (World Bank, Washington DC, USA) on Android tablets, trained enumerators collected two datasets via face‐to‐face interviews: (1) anthropometric screening of ~28,000 children (6–59 months); and (2) in‐depth household, caregiver, and child data were collected through structured surveys and interviews from caregivers of 684 children with SAM, including those under SAM treatment, of whom 486 were aged 6–23 months.

### 2.3 Data Collection and Measurements

The primary study outcomes were IYCF indicators(WHO/UNICEF 2021): (1) breastfeeding status; (2) timely introduction of solid, semi‐solid, or soft foods; (3) MDD (≥ 5 out of 8 food groups); (4) MMF (number of times children receive solid/semi‐solid/soft foods daily according to age); (5) MAD (meeting both diversity and frequency); and the secondary indicators (6) consumption of egg and/or flesh foods; (7) consumption of sweet beverages (Commercially‐produced sweetened drinks, 100% fruit juice drinks, or home‐made sweetened drinks); (8) unhealthy foods (consumption of sentinel unhealthy foods Sweet snacks, fried/salty snacks, or foods high in free sugars, saturated/trans fats, or salt); (9) zero vegetable/fruit consumption (ZVF; no vegetables/fruits during the previous day). Each IYCF indicator was scored dichotomously (0/1) from 24‐h dietary recall either meeting the minimum threshold or not. All measures adhered to WHO 2010 definitions, enabling standardized assessment of IYCF adequacy in relation to maternal, household, and programmatic factors(WHO, UNICEF 2021). Determinants variables included household (HH) factors such as HH wealth assessed Household wealth was assessed using a wealth index derived from principal component analysis (PCA) of household assets and living‐condition variables, following the Demographic and Health Surveys (DHS) approach. The PCA was applied to a set of binary indicators, including ownership of selected assets (e.g., radio, television, bicycle, motorcycle, car), access to utilities, housing characteristics, and financial and social protection indicators (e.g., bank account, health insurance, safety‐net participation). The first principal component was used to generate household wealth scores for regression analysis and was subsequently categorized into low, medium, and high wealth groups for socio‐demographic description. HH food insecurity assessed using the validated Household Food Insecurity Access Scale (HFIAS). Maternal factors included age, education, and literacy. Maternal knowledge on child nutrition and health was assessed using a standardized 32‐item questionnaire encompassing five domains: breastfeeding, complementary feeding, child health, CMAM and SAM screening. Maternal depression was assessed using the Edinburgh Postnatal Depression Scale (EPDS), a standardized and widely validated 10‐item questionnaire designed to screen for symptoms of perinatal depression(e.g., loss of laughter, excessive guilt, sadness, suicidal thoughts) (Cox et al. 1987). EPDS cores were dichotomized into possible depression (EPDS ≥ 10) and no/less possible depression (EPDS < 10), based on established cut‐offs (Cox et al. 1987). Caregiver community involvement was measured using a 10‐item scale assessing participation in community activities (women's discussion groups on health, nutrition, school problems, and community issues), with higher scores indicating greater involvement. Caregiver mobility freedom was measured using a 10‐item scale assessing permission requirements across daily activities (visiting family, market, health services, community issues), with higher scores indicating greater household restrictions. Caregiver dietary diversity score and MDD were assessed using a 24‐h recall, classifying foods into 10 groups; MDD was defined as consumption of five or more groups. Caregiver decision making autonomy (independent decisions made alone, without spousal/others approval) reported by caregivers across key domains: child feeding practices, healthcare seeking, food/livestock purchasing, and household expenditures. This was scored on a 13‐item scale (higher scores indicating greater autonomy), following validation against standard DHS Women's empowerment indices.), married/living with husband, income activities, stigma perception, child characteristics (age, sex, anthropometry).

The anthropometric measurements were standardized through three training sessions, each involving repeated measurements on a group of 10 children, to ensure accuracy and reliability of the data collected by the enumerator teams both absolute and relative technical measures errors and validations check on each of the anthropometrists. The survey captured IYCF indicators, food security, wealth index, hygiene, child health, and livelihoods. All tools were translated to Amharic and Oromifa, back translated, pretested, and administered after 14‐days intensive training.

### 2.4 Data Management and Quality Control

The data collectors were standardized during the training and all precautions including calibration of the equipment and proper positioning, reading the values and recording were used during data collection. Data were reviewed daily for completeness, and flagged for correction before secure, anonymized upload to a password‐protected server. The survey was piloted in the Manna Woreda and adjustments were made to the questionnaire and the procedures before the main survey.

### 2.5 Data Analysis

Descriptive statistics were used to describe household, caregiver, and child traits by livelihood and woreda. Data were processed and analyzed using Stata version 17. Mean knowledge scores were calculated overall and by woreda. The internal consistency of the maternal knowledge score was checked using Cronbach's alpha. Bivariate and multivariable mixed effects Poisson regression models with robust variance estimation accounting for clustering were used assess the association between factors and IYCF indicators, with woreda as a fixed effect and Kebele as a random effect. Collinearity was assessed by inspecting variance inflation factors (VIF) for all factors. Household, caregiver‐ and child‐level factors with *p* < 0.25 in bivariate analysis were retained as candidate factors for the multivariable models.

The level of IYCF indicators in pastoralist and agrarian environments was assessed using nine IYCF indicators, descriptively. However, regression analysis was conducted for three IYCF outcomes using only three of these indicators. In children aged 6–23 months, there are three core indicators: MDD, MMF, and MAD (WHO/UNICEF 2021). However, MAD is not included for regression because it is already a combination of MDD and MMF. Additionally, we examined consumption of eggs and flesh foods, an IYCF indicator strongly associated with child nutritional status and SAM risk (Hailu et al. 2022). There is evidence that children who eat meat and eggs absorb more of the different nutrients necessary for healthy linear growth (Papanikolaou and Fulgoni 2018). Unhealthy feeding practices including consumption of sweetened beverages, unhealthy processed foods, and absence of fruits and vegetables were excluded from determinant analysis due to their limited relevance for SAM children (Pries et al. 2019), the consumption of these food groups predominantly observed in urban settings, while being rarely reported or accessible in rural and pastoralist communities (Malik and Hu 2022).

### 2.6 Ethics Statement

Ethical approval was obtained from the Ethiopian Midwives Association IRB (Reference EMwA‐IRB‐SOP‐/010/4‐24), in compliance with national research ethics standards. All anthropometric measurements on children followed WHO protocols with ethical safeguards: measurements were taken gently by trained professionals, children were never forced and parental oral consent was obtained. All children identified as SAM through screening were immediately referred to the nearest CMAM OTP per national guidelines. Prior to fieldwork, local health authorities and community leaders were briefed on the study's purpose to ensure transparency and community buy‐in. Written informed consent was secured from all caregivers prior to in‐depth interviews; oral consent was obtained for rapid anthropometric screening. The study protocol is registered on clinicaltrials.gov under NCT06380504.

## 3 Results

### 3.1 Socio‐Demographic Characteristics of Children Aged 6–23 Months

All invited caregivers provided written informed consent for the survey interviews, with no refusals. Caregivers of children with SAM and under SAM treatment aged 6–23 months in pastoralist and agrarian settings were mostly biological mothers (96%), with an average age of 28.8 years (±.0 SD). Most caregivers had income generating activities (64%). Children had an average age of 12.3 months (±0.2SD) (Table 1).

**Table 1:** Socioeconomic and demographic characteristics of infants and young children aged 6–23 months in agrarian and pastoralist settings (*n* = 486).

Variables	Agrarian	Pastoralist	Total
	*n*(%)/Mean ± SD	*n*(%)/Mean ± SD	*n*(%)/Mean ± SD
**Household characteristics**	***n*** = **432**	***n*** = **54**	***n*** = **486**
Wealth index			
Low	107 (24.8)	48 (88.9)	154 (31.7)
Medium	157 (36.3)	5 (9.3)	163 (33.5)
High	168 (38.9)	1 (1.9)	169 (34.8)
Food insecurity	280 (64.3)	42 (77.8)	321 (66.0)
Improved water source	301 (69.7)	22 (40.7)	324 (66.7)
Improved water treatment used	24 (5.6)	13 (24.1)	38 (7.8)
Hand washing station with soap available	137 (31.7)	1 (1.9)	139 (28.6)
Number of under five children	1.8 ± 0.7	2.0 ± 0.8	1.8 ± 0.7
Number of adopted under five children	1.4 ± 1.6	1.4 ± 1.4	1.4 ± 1.6
Total number of live‐born children	3.9 ± 2.4	4.0 ± 2.3	3.8 ± 2.3
**Caregiver characteristics**	***n*** = **432**	***n*** = **54**	***n*** = **486**
Knowledge	9.5 ± 3.9	10.5 ± 4.1	9.5 ± 3.9
Biological mother	415 (96.1)	54 (100.0)	469 (96.5)
Age, yr	28.8 ± 7.1	27.0 ± 5.6	28.8 ± 7.0
Married/living together with spouse	402 (93.1)	53 (98.1)	455 (93.6)
Currently pregnant	39 (9.0)	3 (5.6)	42 (8.6)
Attended school	128 (29.6)	16 (29.6)	145 (29.8)
Had income generating activity	285 (66.0)	23 (42.6)	308 (63.4)
Literate	190 (44.0)	40 (74.1)	231 (47.5)
Perceived lack of time (0–10)			
Low	143 (33.1)	18 (33.3)	160 (32.9)
Medium	140 (32.4)	22 (40.7)	163 (33.5)
High	149 (34.5)	14 (25.9)	163 (33.5)
Encourage child to eat	349 (80.8)	36 (66.7)	385 (79.2)
Had decision‐making autonomy (0–13)			
Less empowered	159 (36.8)	17 (31.5)	175 (36.0)
Moderately empowered	126 (29.2)	19 (35.2)	145 (29.8)
Highly empowered	147 (34.0)	18 (33.3)	166 (34.2)
Community involvement(0–10)	1.0 ± 1.7	2.0 ± 2.2	1.1 ± 1.8
Possible depression (EPDS ≥ 10)	182 (42.1)	21 (38.9)	202 (41.6)
Mobility restriction			
Low	145 (33.6)	16 (29.6)	161 (33.1)
Medium	143 (33.1)	20 (37.0)	163 (33.5)
High	144 (33.3)	18 (33.3)	162 (33.3)
Perceived stigma regarding SAM	62 (14.4)	4 (7.4)	66 (13.6)
MDD (#FG ≥ 5)	91 (21.1)	13 (24.1)	103 (21.2)
Total number of pregnancies in lifetime	4.3 ± 2.5	4.3 ± 2.5	4.2 ± 2.4
Frequently absent from child	19 (4.4)	0 (0.0)	19 (3.9)
**Child characteristics**	***n*** = **432**	***n*** = **54**	***n*** = **486**
Age (months)	12.3 ± 4.1	11.8 ± 4.7	12.32 ± 0.20
Female child	264 (60.4)	31 (57.4)	295 (60.1)
HAZ score	−2.7 ± 1.4	−2.2 ± 1.3	−2.8 ± 1.5
Severe stunting (HAZ < −3)	174 (40.3)	13 (24.1)	187 (38.5)
WAZ –score	−2.8 ± 1.1	−2.6 ± 1.4	−3.0 ± 1.1
Severe underweight (WAZ < –3)	181 (44.7)	21 (42.9)	202 (44.5)
WHZ‐score	−1.8 ± 1.1	−1.9 ± 1.3	−1.9 ± 1.3
Sever wasting(WHZ < –3)	71 (16.2)	15 (27.8)	86 (17.5)
Child edema	61 (14.0)	22 (40.7)	83 (16.9)
Child enrolled in SAM OTP^a^	54 (12.5)	13 (24.1)	67 (13.8)
MUAC < 115 mm	326 (74.6)	21 (38.9)	347 (70.7)

Abbreviations: EPDS = Edinburgh Postnatal Depression Scale, FG = Food Group, HAZ = Length/height‐for‐age Z‐score, MDD = minimum dietary diversity, MUAC = mid upper arm circumference, SAM = Sever acute malnutrition, WAZ = Weight‐for‐age Z‐score, WHZ = Weight‐for‐height Z‐score.

*Note:* Data are mean ± SD or *n* (%). Mixed‐effects Poisson regression models with robust estimation of standard errors with woreda as fixed effect and a random intercept to account for clustering by kebele were used for the analysis.

^a^ Child enrolled in SAM OTP was based on three criteria: (i) the caregiver reporting that the child was enrolled in SAM OTP at the time of the survey, (ii) the caregiver reporting that the child was given RUTF in the last three days, and (iii) the caregiver able to show at least one full sachet of RUTF or at least two empty RUTF sachets.

### 3.2 IYCF Indicators of Children Aged 6–23 Months

In both pastoralist and agrarian settings, continued breastfeeding rates among children aged 12–23 months were high (99.5% vs. 100.0%). Children aged 6‐8months timely introduction of semi‐solid foods was nearly universal (> 92%). Children aged 6–23 months had low MDD overall, but higher achievement in pastoralist (31.5%) versus agrarian settings (19.0%; *p* = 0.012). There was no significant difference in MMF between agrarian (53.9%) and pastoralist children (51.9%; *p* = 0.844). Egg/flesh food consumption was lower in pastoralist (5.6%) versus agrarian children (16.9%; *p* = 0.085) (Table 2).

**Table 2:** IYCF indicator among Children Aged 6–23 months in agrarian and pastoralist settings.

IYCF indicators	Agrarian	Pastoralist	Total	*p*‐value^a^
	*n* (%),	*n* (%)	*n* (%)	
	*n* = 432	*n* = 54	*n* = 486	
Continued breastfeeding (12–23 mo)	208 (99.5)	19 (100)	228 (99.6)	—c
Timely introduction of (semi)solid and soft foods (6–8mo)^b^	88 (91.7)	22 (100)	118 (93.2)	—c
Minimum dietary diversity (≥ 5 food groups) (6–23 mo)	82 (19.0)	17 (31.5)	99 (20.4)	0.012
Minimum meal frequency (6–23 mo)	233 (53.9)	28 (51.9)	261 (53.7)	0.844
Minimum acceptable diet (6–23 mo)	55 (12.7)	7 (13.0)	62 (12.8)	0.896
Egg and/or flesh food consumption (6–23 mo)	73 (16.9)	3 (5.6)	76 (15.6)	0.085
Sweet beverage consumption (6–23 mo)	133 (30.8)	8 (14.8)	141 (29.0)	0.017
Unhealthy food consumption (6–23 mo)	49 (11.3)	3 (5.6)	52 (10.7)	0.324
Zero vegetable or fruit consumption (6–23 mo)	222 (51.4)	17 (31.5)	239 (49.2)	0.050

*Note:* Data are *n* (%).

^a^ P‐values were estimated using a mixed‐effects Poisson regression model, with woreda as a fixed effect and a random intercept at the kebele level, comparing pastoralist with agrarian woredas (reference group).

^b^ Data on timely introduction of (semi)‐solid and soft foods (6–8 mo) are based on a smaller subsample (*n* = 118).

^c^ No p‐value was estimated for continued breastfeeding (12–23 mo) and timely introduction of (semi)solid and soft foods (6–8 mo.), because woreda predicted these indicator completely.

### 3.3 Food Group Consumed Among Children Aged 6–23 Months

**Figure 1:**
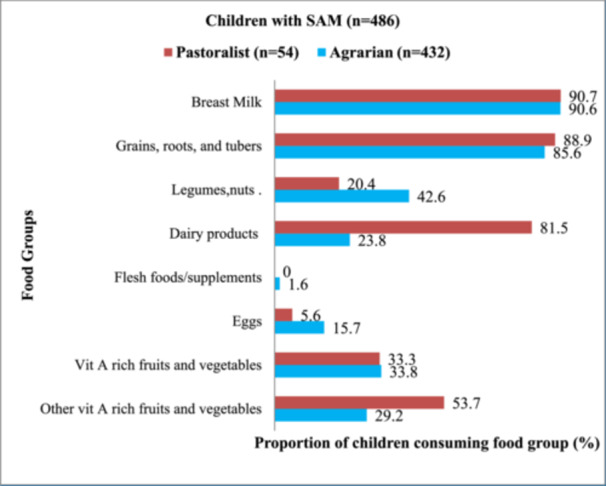
Proportion of children with SAM and under SAM treatment aged 6–23 months (*n* = 486) consuming IYCF food groups, agrarian and pastoralist settings, Ethiopia, colors representing pastoralist and agrarian households.

Among SAM children (6–23 months) in pastoralist and agrarian settings, breast milk and grains/tubers were commonly consumed (> 89%) (Figure 1). Pastoralist children had higher dairy and vitamin A‐rich fruit/vegetable intake, while agrarian children consumed more legumes and nuts. Egg and flesh food intake was low, especially in pastoralist areas (Figure 1).

### 3.4 Factors Associated With Minimum Meal Frequency of Children Aged 6–23

Caregiver literacy was associated with increased likelihood of children achieving MMF in multivariable analysis (RR = 1.21; 95% CI: 1.02–1.43, *p* = 0.030) (Table 3). Conversely, possible caregiver depression was associated with reduced likelihood of children meeting MMF (RR = 0.97; 95% CI: 0.95–0.99; *p* = 0.003).

**Table 3:** Multivariable analysis of factors associated with minimum meal frequency among children with SAM and under SAM treatment aged 6–23 months in pastoralist and agrarian settings in Ethiopia.

Variables	Minimum meal frequency		Bivariate analysis		Multivariable analysis	
	No	Yes				
	*n* = 225	*n* = 261	IRR (95% CI)	*p*‐value	IRR (95%CI)	*p*‐value
**Household characteristics**						
Wealth index						
Low	73 (32.4)	81 (31.0)	1.10 (0.55–2.17)	0.791	—	
Medium	72 (32.0)	91 (34.9)				
High	80 (35.6)	89 (34.1)				
Food insecurity	150 (66.7)	171 (65.5)	0.98 (0.80–1.08)	0.832	—	
Improved water source	153 (68.0)	171 (65.5)	0.94 (0.81–1.10)	0.465		
Improved water treatment used	13 (5.8)	25 (9.6)	1.28 (0.99–1.65)	0.056	1.23 (0.94–1.61)	0.151
Hand washing station with soap available	56 (24.9)	83 (31.8)	1.17 (0.97–1.40)	0.099	1.18 (1.01–1.38)	0.057
Number of under five children	1.8 ± 0.7	1.8 ± 0.7	1.08 (0.96–1.22)	0.213	1.07 (0.95–1.21)	0.300
Number of adopted under five children	1.4 ± 1.4	1.4 ± 1.8	1.01 (0.96–1.06)	0.686		
Total number of live‐born children	3.7 ± 2.2	4.1 ± 2.4	1.03 (0.99–1.07)	0.138	0.99 (0.89–1.10)	0.889
**Caregiver characteristics**					—	
Knowledge	9.5 ± 4.1	9.7 ± 3.8	1.01 (0.99–1.03)	0.455	—	
Age, year	28.4 ± 7.0	28.9 ± 6.9	1.00 (0.99–1.02)	0.451	—	
Married/living together with spouse	214 (95.1)	241 (92.3)	0.82 (0.64–1.07)	0.262	—	
Currently pregnant	20 (8.9)	22 (8.4)	0.97 (0.66–1.42)	0.881	—	
Attended school	64 (28.4)	81 (31.0)	1.06 (0.86–1.31)	0.588	—	
Had income generating activity	151 (67.1)	157 (60.2)	0.87 (0.67–1.12)	0.279		
Literate	98 (43.6)	133 (51.0)	1.16 (0.98–1.37)	0.088	1.21 (1.02–1.43)	0.030
Perceived lack of time (0–10)	4.1 ± 2.4	3.6 ± 2.5	0.96 (0.91–1.01)	0.137	0.97 (0.91–1.02)	0.216
Encourage child to eat	173 (76.9)		1.13 (0.95–1.36)	0.177	1.15 (0.96–1.38)	0.119
Had decision‐making autonomy (0–13)	6.2 ± 3.2	5.7 ± 3.1	0.98 (0.95–1.01)	0.165	—	
Community involvement (0–10)	1.2 ± 1.7	1.1 ± 1.8	0.99 (0.93–1.04)	0.659		
Possible depression (EPDS ≥ 10)	119 (52.9)	83 (31.8)	0.96 (0.94–0.98)	< 0.001	0.97 (0.95–0.99)	0.003
Mobility restriction (0–20)	6.8 ± 4.4	6.3 ± 4.4	0.99 (0.97–1.01)	0.330	—	
Biological mother	216 (96.0)	253 (96.9)	1.15 (0.71–1.86)	0.561	—	
Perceived stigma regarding SAM	29 (12.9)	37 (14.2)	1.05 (0.84–1.31)	0.686	—	
MDD (#FG ≥ 5)	48 (21.3)	55 (21.1)	0.99 (0.77–1.29)	0.958		
Total number of pregnancies in lifetime	4.1 ± 2.4	4.5 ± 2.6	1.03 (0.99–1.07)	0.138	1.04 (0.94–1.15)	0.456
**Child characteristics**					**—**	
Age, months	12.4 ± 4.4	12.2 ± 4.2	1.00 (0.98–1.01)	0.602		
Female	144 (64.0)	146 (55.9)	0.84 (0.69–1.02)	0.072	0.87 (0.72–1.05)	0.151
Pastoralist versus agrarian setting			0.96 (0.64–1.42)	0.844		

*Note:* Mixed‐effects Poisson regression models with robust estimation of standard errors with woreda as a fixed effect and a random intercept to account for clustering by kebele.

Abbreviations: CI = confidence interval, EPDS = Edinburgh Postnatal Depression Scale, FG = food group, MDD = minimum dietary diversity, SAM= severe acute malnutrition, RR = relative risks.

### 3.5 Factors Associated With Minimum Dietary Diversity of Children Aged 6–23 Months

The multivariable analysis showed that household use of an improved water source was associated with higher likelihood of achieving MDD among children aged 6–23 months with SAM and under SAM treatment (RR = 1.59, 95% CI: 1.06–2.38; *p* = 0.037). Caregiver literacy was associated with a higher likelihood of achieving MDD (RR = 2.04, 95% CI: 1.25–3.34; *p* = 0.002). Caregiver MDD was also positively associated with child MDD (RR = 2.21, 95% CI: 1.60–3.05; *p* < 0.001). In addition, older child age was associated with a greater likelihood of achieving MDD (RR = 1.05, 95% CI: 1.02–1.08; *p* ≤ 0.001), and children in pastoralist settings were more likely to meet MDD compared to those in agrarian areas (RR = 1.62, 95% CI: 1.07–2.44; *p* = 0.014). In contrast, higher levels of caregiver depressive symptoms were associated with a lower likelihood of children achieving MDD (RR = 0.94, 95% CI: 0.90–0.97; *p* ≤ 0.001). Greater maternal mobility restrictions was also modestly associated with a lower likelihood of achieving MDD (RR = 0.94, 95% CI: 0.89–1.00; *p* = 0.025). Additionally, household food insecurity was associated with a lower likelihood of achieving MDD, although this association was borderline significant (RR = 0.64, 95% CI: 0.41–1.01; *p* = 0.054) Table 4.

**Table 4:** Multivariable analysis of factors associated with minimum dietary diversity among children with SAM and under SAM treatment aged 6–23 months pastoralist and agrarian settings in Ethiopian.

Variables	Minimum dietary diversity		Bivariate analysis		Multivariable analysis	
	No	Yes				
	*n* = 387	*n* = 99	IRR (95% CI)	*p*‐value	IRR (95%CI)	*p*‐value
**Household characteristics**						
Wealth index			4.82 (1.35–17.03)	0.015	1.77 (0.45–6.91)	0.426
Low	127 (32.8)	27 (27.3)				
Medium	136 (35.1)	27 (27.3)			—	
High	124 (32.0)	45 (45.5)			—	
Food insecurity	275 (71.1)	46 (46.5)	0.42 (0.27–0.65)	< 0.001	0.64 (0.41–1.01)	0.054
Improved water source	249 (64.3)	75 (75.8)	1.73 (1.14–2.64)	0.010	1.59 (1.06–2.38)	0.037
Improved water treatment used	25 (6.5)	13 (13.1)	1.59 (1.02–2.48)	0.039	1.06 (0.79–1.43)	0.824
Hand washing station with soap available	107 (27.6)	32 (32.3)	1.37 (0.85–2.19)	0.195	0.76 (0.51–1.16)	0.124
Number of under five children	1.8 ± 0.7	1.9 ± 0.6	1.37 (0.92–1.44)	0.204	1.00 (0.80–1.24)	0.885
Number of adopted under five children	1.4 ± 1.6	1.5 ± 1.7	1.04 (0.94–1.16)	0.455	—	
Total number of live‐born children	3.9 ± 2.4	4.0 ± 2.3	1.01 (0.94–1.09)	0.699	—	
**Caregiver characteristics**					**—**	
Knowledge	9.3 ± 3.9	11.0 ± 4.1	1.08 (1.03–1.13)	0.002	1.03 (0.98–1.07)	0.225
Age, year	28.7 ± 7.3	28.3 ± 5.2	0.99 (0.97–1.02)	0.627		
Married/living together with spouse	358 (92.5)	97 (98.0)	3.11 (0.82–11.50)	0.091	2.39 (0.85–6.74)	0.109
Currently pregnant	33 (8.5)	9 (9.1)	1.07 (0.69–1.65)	0.777	—	
Attended school	102 (26.4)	43 (43.4)	1.79 (1.21–2.74)	0.004	1.04 (0.71–1.53)	0.873
Had income generating activity	249 (64.3)	59 (59.6)	0.90 (0.57–1.42)	0.649		
Literate	161 (41.6)	70 (70.7)	2.58 (1.67–3.98)	< 0.001	2.04 (1.25–3.34)	0.002
Perceived lack of time (0‐10)	3.7 ± 2.4	4.3 ± 2.6	1.07 (0.99–1.17)	0.092	1.05 (0.98–1.12)	0.14
Encouraged child to eat	306 (79.1)	79 (79.8)	1.09 (0.73–1.64)	0.672	—	
Community involvement (0‐10)	1.1 ± 1.7	1.3 ± 2.1	1.03 (0.92–1.16)	0.631		
Possible depression (EPDS ≥ 10)	168 (43.4)	34 (34.3)	0.95 (0.92–0.99)	0.011	0.94 (0.91–0.97)	< 0.001
Mobility restriction (0‐10)	6.8 ± 4.6	5.7 ± 3.9	0.95 (0.91–1.00)	0.035	0.94 (0.89–1.01)	0.025
Biological mother	372 (96.1)	97 (98.0)	1.63 (0.43–6.20)	0.475	—	
Perceived stigma regarding SAM	50 (12.9)	16 (16.2)	1.27 (0.75–2.16)	0.370	—	
MDD (#FG ≥ 5)	64 (16.5)	39 (39.4)	2.41 (1.74–3.33)	< 0.001	2.21 (1.60–3.05)	< 0.001
Total number of pregnancies in lifetime	4.3 ± 2.6	4.3 ± 2.3	1.00 (0.94–1.06)	0.962	—	
**Child characteristics**						
Age, month	12.0 ± 4.4	13.4 ± 3.9	1.06 (1.03–1.09)	< 0.001	1.05 (1.02–1.08)	< 0.001
Female	226 (58.4)	64 (64.0)	1.18 (0.81–1.74)	0.349		
Pastoralist versus agrarian setting			1.66 (1.12–2.47)	0.012	1.62 (1.07–2.44)	0.014

*Note:* Mixed‐effects Poisson regression models with robust estimation of standard errors with woreda as fixed effect and a random intercept to account for clustering by kebele.

Abbreviations: CI = confidence Interval, EPDS = Edinburgh Postnatal Depression Scale, FG = food group, MDD = minimum dietary diversity, RR = relative risk, SAM = severe acute malnutrition.

### 3.6 Factors Associated With Egg and Flesh Food Consumption in Children Aged 6–23

The multivariable analysis revealed several factors that were associated with children consumed eggs and/or flesh foods (Table 5). Children were more likely to eat these nutrient‐rich foods when their caregivers were more actively involved in the community (RR = 1.12; 95% CI: 1–1.26; *p* = 0.038) and when caregivers themselves met MDD standards (RR = 1.67; 95% CI: 1.1–2.53; *p* = 0.043). Older children also had a higher likelihood of consumption (RR = 1.08; 95% CI: 1.05–1.12; *p* < 0.001). On the other hand, children in pastoralist settings were significantly less likely to consume these foods compared to those in agrarian settings (RR = 0.36; 95% CI: 0.13–0.97; *p* = 0.038).

**Table 5:** Multivariable analysis of factors associated with consumption of egg and flesh foods among children with SAM and under SAM treatment aged 6–23 months pastoralist and agrarian settings in Ethiopian.

Variables	Egg and flesh food		Bivariate analysis		Multivariable analysis	
	No	Yes				
	*n* = 410	*n* = 76	IRR (95%CI)	*p*‐value	IRR (95%CI)	*p*‐value
**Household characteristics**						
Wealth index			4.55 (1.31–15.76)	0.017	1.47 (0.28–7.05)	0.682
Low	141 (34.4)	13 (17.1)				
Medium	139 (33.9)	24 (31.6)			—	
High	130 (31.7)	39 (51.3)				
Food insecurity	282 (68.8)	39 (51.3)	0.57 (0.36–0.92)	0.023	0.71 (0.41–1.21)	0.208
Improved water source	264 (64.4)	60 (78.9)	1.71 (0.98–2.99)	0.061	1.35 (0.78–2.34)	0.285
Improved water treatment used	31 (7.6)	7 (9.2)	1.48 (0.68–3.24)	0.326	—	
Hand washing station with soap available	112 (27.3)	27 (35.5)	1.20 (0.76–1.91)	0.431	—	
Number of under‐five children^a^	1.8 ± 0.7	1.9 ± 0.8	1.37 (1.04–1.81)	0.026		
Number of adopted under five children	1.4 ± 1.6	1.6 ± 1.5	1.05 (0.96–1.13)	0.278	—	
Total number of live‐born children	3.9 ± 2.3	4.1 ± 2.5	1.04 (0.96–1.13)	0.299	—	
**Caregiver characteristics**					**—**	
Knowledge	9.4 ± 3.9	10.6 ± 4.2	1.06 (1.00–1.12)	0.033	1.03 (0.96–1.08)	0.634
Age, yr	28.6 ± 7.1	28.6 ± 5.9	1.00(0.97–1.03)	0.905		
Married/living together with spouse	382 (93.2)	73 (96.1)	1.81 (0.74–4.39)	0.192	1.26 (0.56–2.86)	0.575
Currently pregnant	34 (8.3)	8 (10.5)	1.22 (0.66–2.28)	0.528		
Attended school	115 (28.0)	30 (39.5)	1.58 (0.99–2.54)	0.058	1.29 (0.74–2.27)	0.368
Had income generating activity	261 (63.7)	47 (61.8)	0.88 (0.49–1.58)	0.656		
Literate	187 (45.6)	44 (57.9)	1.67 (1.10–2.55)	0.016	1.22 (0.75–1.98)	0.434
Perceived lack of time (0‐10)	3.8 ± 2.5	4.0 ± 2.4	1.03 (0.96–1.11)	0.371		
Encourages child to eat	318 (77.6)	67 (88.2)	1.99 (0.78–5.10)	0.153	2.27 (0.93–5.05)	0.073
Decision power (0‐13)	5.9 ± 3.2	6.3 ± 3.3	1.04 (0.97–1.11)	0.286		
Community involvement (0‐10)	1.0 ± 1.6	1.6 ± 2.1	1.15 (1.01–1.30)	0.028	1.13 (1.01–1.26)	0.038
Possible depression (EPDS ≥ 10)	167 (40.7)	35 (46.1)	1.01 (0.97–1.06)	0.577	—	
Mobility restriction (0‐10)	6.4 ± 4.6	6.9 ± 3.7	1.02 (0.98–1.06)	0.383	—	
Biological mother	395 (96.3)	74 (97.4)	1.45 (0.39–5.41)	0.584	—	
Perceived stigma regarding SAM	52 (12.7)	14 (18.4)	1.38 (0.90–2.13)	0.144	—	
MDD (#FG ≥ 5)	77 (18.8)	26 (34.2)	1.95 (1.25–3.04)	0.003	1.56 (1.01–2.40)	0.043
Total number of pregnancies in lifetime	4.3 ± 2.5	4.4 ± 2.5	1.02 (0.95–1.11)	0.546	—	
**Child characteristics**					**—**	
Age, months	12.0 ± 4.2	14.1 ± 4.4	1.09 (1.05–1.13)	< 0.001	1.08 (1.05–1.12)	< 0.001
Female	244 (59.5)	46 (60.5)	0.97 (0.64–1.45)	0.868		
Pastoralist versus agrarian setting			0.33 (0.12–1.11)	0.085	0.35 (0.13–0.94)	0.038

*Note:* Mixed‐effects Poisson regression models with robust estimation of standard errors with woreda as fixed effect and a random intercept to account for clustering by kebele.

Abbreviations: CI = confidence interval, EPDS = Edinburgh Postnatal Depression Scale, FG = food group, MDD = minimum dietary diversity, RR = relative risk, SAM = severe acute malnutrition.

^a^ The variable “number of under‐five children in the household” was excluded from the final regression model because its inclusion caused coefficient instability in the model when comparing regression estimates.

## 4 Discussion

This study objectively explored IYCF indicators among children with SAM and under SAM treatment aged 6–23 months in pastoralist and agrarian settings of Ethiopia and assessed which household, caregiver, and child‐level determinants were associated with a subset of these indicators.

Pastoralist children with SAM and under SAM treatment had a higher MDD prevalence (31.5%) than agrarian children (19.0%; *p* = 0.012). This difference stems primarily from greater dairy intake, especially milk and milk products from cattle, goats, and camels among pastoralist children during the rainy/wet season. This pattern aligns with prior research linking pastoralists' elevated MDD to seasonal dairy consumption (Potts et al. 2019). and is consistent with findings from West Shewa and Somali regions, where wet‐season dairy boosts dietary diversity (Belete et al. 2022; Sadler, K. & Catley, A. 2009). Pastoralist children with SAM and under SAM treatment also consumed more other vegetables (particularly leafy and wild/semi‐wild greens) and locally available wild fruits, contributing to their higher diversity in the “other vegetables and fruits” food group. This is supported by evidence from eastern Ethiopia, where wild edible plants serve as survival foods and dietary supplements during shortages (Tahir et al. 2023).

The prevalence of MMF was generally low and similar across all children with SAM and under SAM treatment (52%–55%) aligning closely with findings reported for the Afar Region at 43.8% (Wuneh et al. 2019), national estimates of 55.9% (Melak et al. 2024), and estimates of the Jimma zone of 52.9% (Abafita 2025). This suggests that children with SAM and under SAM treatment in our study were fed with similar minimal frequency as children not suffering from SAM from other studies.

Pastoralist study children showed markedly lower egg and flesh food consumption compared to agrarian peers (5.6% vs. 16.9%; *p* = 0.085) likely reflecting sociocultural practices and economic priorities. Eggs and flesh foods in pastoralist communities are often sold or consumed by adults rather than young children, shaped by a lack of poultry farms, cultural norms and livelihoods that prioritize income over young child feeding (Sewenet and Schwarcz 2021). In rural Ethiopian agrarian districts, egg consumption remains modest (16.4%) and flesh food intake very low (2.3%) among children aged 6–23 months (Daba et al. 2024).

Pastoralist study children consumed fewer unhealthy foods and sweet beverages (14.8% vs. 30.8%, *p* = 0.017) and had lower prevalence of zero fruit and vegetable intake (31.5% vs. 51.4%, *p* = 0.050) compared to children from the agrarian woreda. This finding suggests a limited nutrition transition in pastoralist areas, likely related to geographic isolation and poor market access that constrain the availability of ultra‐processed foods compared with agrarian woredas. The high prevalence of zero fruit and vegetable consumption in the agrarian setting was striking perhaps not entirely unexpected, given that national rates in Ethiopia are even higher, reaching an alarming 69.3% (Semagn and Abubakari 2023) and underscores how deeply entrenched low fruit and vegetable intake remains in the general child population.

Caregiver literacy was associated with higher child MMF. This may reflect greater awareness of appropriate feeding practices among literate caregivers, enabling better adherence to appropriate feeding. Supporting this, studies from Ethiopia (Mekonnen et al. 2017), The Gambia (Terefe et al. 2023), Uganda (Ickes et al. 2015), Tanzania (Millanzi et al. 2023), and sub‐Saharan Africa(SSA) (Tebeje et al. 2024), have also consistently shown that maternal literacy consistently correlates with more frequent child feeding and better complementary feeding knowledge (Abeshu et al. 2016). Maternal depression symptoms (loss of laughter, excessive guilt, worry/panic, feeling overwhelmed, sleep disturbance, sadness, uncontrollable crying, suicidal thoughts) were associated with a lower likelihood of children meeting MMF. This underscores how mental health disrupts feeding regularity, consistent with findings from a Norwegian study linking maternal depression to fewer meals and irregular mealtimes (Helle et al. 2024). These mental health challenges also predicted less responsive feeding behaviors (Anato et al. 2020; Haycraft et al. 2013; Helle et al. 2024) and reduced maternal involvement during meals, and more negative mealtime interactions (Hurley et al. 2008; McCurdy et al. 2014).

Our study found that literate caregivers were more likely to have children with SAM and under SAM treatment who met MDD, likely because caregiver literacy improves comprehension of nutrition education and health advice. This relation is well‐established across multiple settings as reported by a study in 31 SSA countries (Paulo et al. 2024) and within Ethiopia (Moga Lencha et al. 2022; Solomon et al. 2017). Possible caregiver depression was negatively associated with MDD, consistent with studies from Kenya and Nepal showing that children from depressed mothers were less likely to meet MDD (Kalam et al. 2025; Miller et al. 2021). Caregiver mobility restriction showed a small negative association with children with SAM and under SAM treatment achieving MDD. Reflections from our findings suggest dual pathways through which restricted mobility perpetuates poor dietary diversity: time trade‐offs (limiting market access and childcare time) and information gaps (reduced nutrition knowledge from social/health networks) even when basic foods are available. This was supported by a study in Tanzania (Galiè et al. 2019). Caregivers who met MDD themselves had children who more often met MDD, reflecting shared household food environments supported by Ghanaian evidence where maternal MDD strongly predicted child MDD (Amugsi et al. 2015). Access to improved water sources was also linked to higher MDD, possibly by reducing illness burden and enabling food preparation hygiene. Similar findings in China and Ethiopia confirm that Unimproved water constrains dietary diversity via disease burden, limited food processing, and lower SES(socioeconomic status), reinforcing the idea that water access is part of a wider socioeconomic and food‐system pathway (Abuye et al. 2024; Gao et al. 2022; Roba et al. 2024).

Older children (12–23 months) showed higher MDD due to greater solid food consumption capacity, while younger infants (6–8 months) remained limited to liquids/semi‐solid porridges consistent with Ethiopian findings (Molla et al. 2021). Finally, children in pastoralist settings were associated with higher MDD. This may reflect their higher dietary diversity than children in agrarian areas, possibly due to more consistent access to nutrient‐rich ASF such as milk and other dairy products. This finding is consistent with a study in Ethiopia (DHS 2011). This finding, though under‐studied, is consistent with the findings from Uganda, where pastoralists children had a more diverse diet than agrarian households (Mayanja et al. 2015).

More community activity involvement of caregivers was associated with increased egg and flesh food consumption among children aged 6–23 months with SAM and under SAM treatment. This finding may suggest that more exchanges with other caregivers may have improved caregiver's knowledge and attitude towards feeding their children ASF. This was consistent with Nigerian findings on nutrition education benefits (Flax et al. 2022). Notably, caregivers who themselves met the MDD were more likely to feed their children eggs and flesh foods. This finding aligns with research from Bangladesh, Vietnam, and Ethiopia, which showed that caregivers who eat diverse diets were more likely to feed their children more nutrient‐rich foods (Nguyen et al. 2013). We also observed that older children were more likely to consume eggs or flesh foods, a finding supported by evidence from other SSA countries attributing this pattern to the transition from predominant breastfeeding to more solid complementary foods, enabling older infants to consume a wider variety of ASF (Hailu et al. 2022). Conversely, children in pastoralist settings showed lower egg and flesh food consumption despite higher MDD, as poultry/egg access remains constrained (DHS 2011).

This study had several strengths and limitations. The data drew from a population‐representative sample of children with SAM and under SAM treatment or enrolled in SAM treatment from two distinct settings, a notoriously difficult group to capture given the low prevalence, requiring the screening of approximately 28,000 children in the case of this study. The sample spanned two distinct woredas, though the pastoralist woreda contributed a notably smaller sample size due to its smaller overall population. Households in pastoralist areas were frequently absent, as families moved with their livestock for grazing and conducted farming activities far from home. For certain IYCF indicators, such as timely introduction of (semi)solid and soft foods and continued breastfeeding, which are assessed within a narrower age range, the analysis had to rely on even smaller subgroups. The association between paternal characteristics and IYCF indicators could not be assessed, as fathers particularly in the pastoralist setting were frequently away from home for the entire day, leaving substantial gaps in paternal data.

These findings support targeted IYCF interventions within SAM OTP services via Social and Behavior Change Communication (SBCC), focusing on complementary feeding timing, dietary diversity, and ASF consumption during and post‐discharge. While SAM children's IYCF practices appear comparable to non‐SAM peers, more intensive support may be needed if future studies confirm worse performance.

## 5 Conclusion

This study assessed IYCF practices among children aged 6–23 months with SAM or under SAM treatment in agrarian and pastoralist settings in Ethiopia. MDD was low and context‐dependent, and egg and flesh food consumption was particularly low among pastoralist children. Caregiver literacy, caregiver MDD, improved water access, older child age, and community engagement were positively associated with selected IYCF practices, while caregiver depression, household food insecurity, and mobility restrictions were linked to poorer practices. Overall, the findings support targeted, setting‐specific interventions within SAM treatment services, including livelihood‐sensitive foods, improved access to eggs, flesh foods, fruits, and vegetables, WaSH improvements, community engagement, caregiver mental support and IYCF counseling within CMAM programs and SBCC.

## Author Contributions

All the authors contributed to: Tefera Belachew, Lieven Huybregts, Mohammed Areb, Alemayehu Haddis in conception. Tefera Belachew, Lieven Huybregts, Mohammed Areb, Alemayehu Haddis designed the research study. Mohammed Areb, Lieven Huybregts, Mariama Touré, Tefera Belachew, Talla Fall in formal analysis, and interpretation. Tefera Belachew, Lieven Huybregts, Mohammed Areb, Bayise Biru, Dessalegn Tamiru, Alemayehu Haddis in Supervision. Mohammed Areb wrote the original paper. Writing‐Review and editing. Tefera Belachew, Lieven Huybregts, Dessalegn Tamiru, Mariama Touré, Talla Fall. All Authors read and approved the final manuscript. Mohammed Areb, took the responsibility of drafting the article and is responsible to submit it.

## Conflicts of Interest

The authors declare no conflicts of interest.

## Data Availability

The data are securely managed and stored on password‐protected servers operated by the Ethiopian Public Health Association (EPHA) and the International Food Policy Research Institute (IFPRI), with access restricted to authorized researchers.
